# Global Research Trends in Innate Lymphoid Cells in the Gut and Kidney: A Bibliometric and Visual Analysis

**DOI:** 10.2174/0118715303409659251128095655

**Published:** 2026-05-21

**Authors:** Xing Zhang, Gengxin Liu, Ziyou Yan

**Affiliations:** 1 School of Traditional Chinese Medicine, Shandong University of Chinese Medicine, Jinan, China;; 2 Department of Traditional Chinese Medicine, Jinan People's Hospital, Jinan, China;; 3 School of Clinical Medicine, Jiangxi University of Traditional Chinese Medicine, Nan Chang, China

**Keywords:** Innate lymphoid cells, kidney, gut, inflammation, immunity, inflammatory bowel disease

## Abstract

**Introduction:**

Innate lymphoid cells (ILCs), critical immune regulators in mucosal tissues, maintain tissue homeostasis, modulate inflammation, and drive immune defense. While their roles in gut and kidney biology are increasingly studied, global research trends and focal points in this field remain largely uncharacterized.

**Methods:**

Using the Web of Science Core Collection (2004–2024), we analyzed 6,835 articles on gut- and kidney-associated ILCs. Bibliometric tools (VOSviewer, CiteSpace) were employed to evaluate publication trends, collaborations, institutional contributions, author influence, journal profiles, and keyword patterns to identify research priorities.

**Results and Discission:**

Annual publications surged from 2004 to 2024, peaking at 622 in 2024. The U.S. (2,145 articles), China (1,539), and Germany (679) dominated output, with Harvard Medical School and Inserm as leading institutions. Gregory F. Sonnenberg and David Artis were the most-cited authors. Key journals included *Frontiers in Immunology*, *Immunity*, and *Journal of Immunology*. Research hotspots centered on inflammatory mechanisms (*e.g.*, inflammatory bowel disease), immunotherapy, and ILC-microbiome crosstalk. Emerging trends emphasized tumor microenvironment interactions, as highlighted by keyword burst analysis.

**Conclusion:**

This study underscores the therapeutic potential of ILCs in inflammation and cancer within the gut and kidney, advocating for interdisciplinary collaboration to advance mechanistic and clinical research.

## 1 INTRODUCTION

Innate lymphoid cells (ILCs) are a subset of lymphocytes that do not express T-cell or B-cell antigen receptors [1]. They are widely distributed in mucosal barrier tissues, such as the skin, gut, and brain, and play critical roles in maintaining tissue homeostasis, regulating inflammatory responses, and participating in immune defense [2]. Based on their transcription factors and cytokine profiles, ILCs are classified into cytotoxic natural killer (NK) cells and four “helper” ILC groups: ILC1, ILC2, ILC3, and lymphoid tissue inducer (LTi) cells [3]. Historically, helper ILCs have been extensively studied and recognized as key regulators of immune responses in various organs and inflammatory conditions. Recent advances in single-cell sequencing and gene-editing technologies have further elucidated the functions of ILCs in the gut and kidney, as well as their associations with diseases. For instance, the regulatory mechanisms of ILCs in inflammatory bowel disease (IBD) and their roles in renal fibrosis have become focal points in immunology and clinical research [4].

In kidney diseases, recent studies have demonstrated that ILC3s (type 3 innate lymphoid cells) play a significant role in renal fibrosis [5]. ILC3s are activated *via* the IL-23-dependent signaling pathway and interact with mononuclear phagocytes (MNPs) to promote renal inflammation and fibrosis. Additionally, ILC3s can exacerbate renal fibrosis through PD-1-mediated activation pathways, revealing the migration routes of ILC3s along the gut-kidney axis and their potential pathogenic mechanisms in kidney diseases [4]. In the gut microenvironment, ILCs interact with commensal microbiota, neurotransmitters, and immune cells to maintain intestinal homeostasis [6]. For example, ILC2s (type 2 innate lymphoid cells) regulate immune responses through bidirectional communication with enteric neurons, providing new insights into the interplay between the gut immune system and the nervous system [7]. Furthermore, the roles of ILC3s in intestinal inflammation and tissue repair have been increasingly elucidated, offering new perspectives on the functional regulation of ILCs across different tissues [4, 8].

Despite these advancements, the global research trends, hotspots, and future directions in the study of ILCs in the gut and kidney remain inadequately summarized. Bibliometric and visual analysis tools, such as CiteSpace and VOSviewer, provide powerful means to systematically review and predict research trends. These tools enable multidimensional analysis of keywords, author collaboration networks, and journal citations, revealing dynamic changes and future directions in the field.

This study aims to employ bibliometric and visual analysis methods to systematically summarize global research trends on gut and kidney ILCs from 2004 to 2024. By providing a comprehensive overview of the current research landscape, this study seeks to offer valuable insights for future research directions. Additionally, it explores the potential applications of ILCs in gut and kidney diseases, providing theoretical support for the development of clinical therapeutic strategies.

## 2 METHODS

### 2.1 Data Collection

We conducted a comprehensive search of the Web of Science Core Collection (WOSCC), including the Science Citation Index Expanded (SCI-EXPANDED) and the Social Sciences Citation Index (SSCI), for articles published between January 1, 2004, and December 31, 2024. The search strategy was as follows: ((((((TS=(ILC)) OR TS=(ILCs)) OR TS=(Innate lymphoid cells)) OR TS=(lymphoid tissue inducer)) OR TS=(LTi)) OR TS=(natural killer cell)) OR TS=(NK cell) AND ((((((TS=(renal)) OR TS=(kidney)) OR TS=(nephropathy)) OR TS=(gut)) OR TS=(Colon)) OR TS=(colorectal)) OR TS=(Rectum)) OR TS=(Intestine]. The Web of Science Core Collection (WOSCC) was selected as the primary data source due to its high reliability, comprehensive coverage, and advanced bibliometric capabilities. WOSCC is a well-established citation database that employs rigorous journal selection criteria, ensuring the inclusion of high-impact, peer-reviewed literature [9]. Compared to other databases such as Scopus and PubMed, WOSCC offers superior citation tracking and indexing, making it the most suitable choice for this study [10]. The search results were manually screened based on article type, language, publication date, and relevance to the research topic to ensure the quality of the included literature. The data extraction process is illustrated in Fig. (**1**).

### 2.2 Bibliometric Analysis

The extracted literature data were imported into Microsoft Excel 2019 to generate a line chart depicting the annual distribution of publications. For visual analysis, VOSviewer (version 1.6.20; Leiden University, Leiden, Netherlands) [11] and CiteSpace (version 6.3.1.0, Drexel University, Philadelphia, PA, USA) [12] were employed to analyze the annual trends, country/region contributions, institutional collaborations, author influence, journal distributions, citation patterns, and keyword co-occurrence in the field of ILC research in the gut and kidney [13]. VOSviewer was used to create network visualization maps, where nodes of the same color represent clusters, connections between nodes indicate collaborations, and node size reflects the number of publications or keyword frequency.

CiteSpace was utilized for cluster analysis and burst detection. Modularity Q and mean silhouette are key evaluation metrics for cluster analysis. Generally, a modularity Q > 0.3 indicates a significant cluster structure, while a mean silhouette > 0.5 or > 0.7 suggests reasonable or highly convincing clustering results [14]. Additionally, burst analysis was performed on keywords and references to identify emerging trends and research hotspots. In CiteSpace, the log-likelihood ratio (LLR) algorithm with default parameters was applied for cluster analysis [15]. For burst detection [8], the following default parameters were used: configuration F(x) = ae^(-ax), 1/a0 = 2.0, a_i/a_(i-1) = 2.0, state number = 2, γ [0,^1^] = 1.0, and minimum duration = 2.

## 3 RESULTS

### 3.1 Publication Volume and Trends

A total of 6,835 articles were included in this study, comprising 4,972 original research articles and 1,863 review articles. The first article in this field was published by Hilde [16], who explored the biological functions and evolutionary significance of mucosal T cells in the intestinal immune system, particularly emphasizing the role of intraepithelial lymphocytes (IELs) in distinguishing self from non-self, preventing pathogen invasion, and maintaining mucosal barrier integrity. The study highlighted that the complexity of the intestinal environment (*e.g.*, the presence of abundant commensal microbiota and food antigens) drives the diversification and refinement of the immune system, enabling it to protect the host while maintaining mucosal barrier integrity. The interaction mechanisms between the intestinal microenvironment and the immune system have provided new perspectives on the balance between immune tolerance and immune responses, making this a highly focused area of research.

As shown in the annual publication trend line chart (Fig. **2**), the number of publications in this field exhibited a steady increase from 2004 to 2024, peaking in 2024 with 622 articles, accounting for 9.1% of the total publications. Notably, a significant surge in publications occurred between 2019 and 2021, followed by a temporary decline in 2023, which may be attributed to the COVID-19 pandemic and adjustments in journal impact factors during this period [17]. The fitted curve function (y = 2E-212e^(0.2436x), with an R^2^value of 0.7467, indicates a good fit, suggesting that ILC research in the gut and kidney continues to attract attention and remains in a phase of active development.

Additionally, we collected basic parameters of the included literature: contributions from 104 countries/regions, 40,730 authors, 6,355 institutions, and 1,293 journals, with 275,297 references cited from 13,845 journals. Among these, 28 articles were cited more than 60 times.

### 3.2 Country/Region Publication and Collaboration Analysis

The included publications originated from 104 countries and regions. The top three countries in terms of publication volume were the United States (2,145 articles, 31.38%), China (1,539 articles, 22.52%), and Germany (679 articles, 9.93%). By setting the minimum document threshold to 82, a total of 20 countries met the criteria. The publication volumes and collaboration networks among these countries were visualized (Fig. **3A**), where larger nodes indicate higher publication volumes, and thicker connecting lines represent stronger collaborative relationships.

From the perspective of international collaborations (Fig. **3B**), the United States and China exhibited the closest partnership, followed by collaborations between the United States and Germany, Japan, and the United Kingdom, indicating a rich network of international cooperation in this field. Analysis of annual publication trends by country (Fig. **3C**) revealed that Germany, France, and Japan were early contributors to this field, while China and Australia have emerged as active contributors in recent years, with China now ranking among the top contributors.

We further summarized the publication and citation metrics for the top five countries (Table **1**). The United States had the highest total citation count (159,978 citations), followed by Germany (41,773 citations). France achieved the highest average citation frequency (85.06 citations per article), followed by the United States (74.58 citations per article), highlighting the significant influence of research from these countries. In comparison, China's research impact remains relatively lower, with an average citation frequency of 26.2, suggesting room for improvement through enhanced international collaboration and innovative research.

### 3.3 Institutional Contributions and Author Analysis

The included publications were affiliated with 6,355 institutions. The top 10 institutions by publication volume are summarized in Table **2**. Harvard Medical School ranked first with 101 publications (1.48% of total publications) and 5,668 citations, yielding an average citation count of 56.32. The French National Institute of Health and Medical Research (Inserm) ranked second with 96 publications (1.40%) and 12,015 citations, achieving an average citation count of 129.09. The National Cancer Institute (NCI) ranked third with 94 publications (1.38%) and 9,154 citations, averaging 97.38 citations per article.

In terms of average citation frequency, Inserm (France) ranked highest with 126.09 citations per article, followed by Harvard Medical School with 100.71 citations per article, underscoring their substantial contributions to the field.

The collaboration network among the top 20 institutions was visualized (Fig. **4A**). The clustering analysis revealed that research in this field remains predominantly focused on domestic collaborations. For instance, Shanghai Jiao Tong University and Fudan University in China exhibited the highest collaboration frequency, followed by collaborations between Zhejiang University, the Chinese Academy of Sciences, and institutions such as the University of Washington and the University of California, San Francisco in the United States. However, international collaborations were relatively limited, highlighting the need for stronger cross-border institutional partnerships to advance research in this field.

Analysis of publication trends over time (Fig. **4B**) showed that Harvard University (United States) was an early contributor, while institutions such as Shanghai Jiao Tong University, Fudan University, and Huazhong University of Science and Technology in China have become increasingly active in recent years.

In this study, we analyzed the top 10 most active authors by publication volume (Table **3**). The results revealed that Gregory F. Sonnenberg, David Artis, and Gerard Eberl were the top three authors, with 30, 25, and 25 publications, respectively. David Artis also had the highest average citation count (328.04 citations per article), followed by Gregory F. Sonnenberg with an average of 182.37 citations per article, indicating their significant influence and recognition in the field. Gregory F. Sonnenberg and David Artis are leading scholars in the fields of gut and immunology, as evidenced by their extensive contributions and high citation rates in innate lymphoid cell (ILC) research. Gregory F. Sonnenberg’s research focuses on elucidating the roles of innate lymphoid cells and the gut microbiota in immunity, inflammation, and cancer. In 2024, his team published a groundbreaking study in Nature, revealing that interleukin-23 (IL-23) suppresses inflammation by activating the cytotoxic T-lymphocyte-associated protein 4 (CTLA4) pathway in ILC3s [18]. This regulatory mechanism is often disrupted in inflammatory bowel disease (IBD), leading to chronic inflammation. This discovery provides new insights into the pathological mechanisms of ILC3s in IBD and offers potential therapeutic strategies. David Artis has been a pioneer in ILC research since publishing the first comprehensive review in this field in 2010. In October 2023, he published an extensive review in Nature Reviews Immunology, delving into the interaction mechanisms between type 2 innate lymphoid cells (ILC2s) and helper T cells in defending against intestinal parasites [19]. We also visualized the collaboration networks among authors with ≥8 publications, revealing 15 distinct clusters (Fig. **4C**). The most frequent collaboration was between Gregory F. Sonnenberg and Gerard Eberl. From a temporal perspective (Fig. **4D**), high-impact authors such as Gregory F. Sonnenberg were most active around 2018, while Chinese authors became more prominent after 2020.

### 3.4 Journal Publication Analysis

Using VOSviewer, we analyzed 1,293 source journals and 13,845 co-cited journals. The top 10 journals by publication volume and citation frequency were summarized in a statistical table (Table **4**), and a cluster analysis visualization (Fig. **5A**) and timeline graph (Fig. **5B**) were generated. The top three journals by publication volume were *Frontiers in Immunology* (460 articles, 6.73%, IF=5.7), *Journal of Immunology* (174 articles, 2.55%, IF=3.6), and *PLOS ONE* (142 articles, 2.08%, IF=2.^9^]. The highest average citation count was achieved by *Immunity* (212.69 citations per article, IF=25.5), followed by *Journal of Immunology* (58.72 citations per article, IF=3.^6^]. Most of these journals are concentrated in the fields of immunology and molecular biology, with a few being multidisciplinary journals, and the majority belong to the JCR Q1 category.

The timeline graph revealed that *Frontiers in Pharmacology* and *Frontiers in Medicine* have gained recent attention, suggesting that the development of therapeutic strategies and pharmacological research targeting ILCs in the gut and kidney has become an emerging hotspot. Co-citation analysis of journals helps identify influential journals in the field, with higher citation frequencies indicating greater relevance. The most frequently co-cited journal was *Immunity* (15,148 citations), followed by *Journal of Immunology* (21,656 citations) and *Nature* (14,693 citations). Notably, *Nature* has the highest impact factor among the cited journals. These findings indicate that *Immunity* is the most influential and widely recognized journal in this field. In November 2024, Liang *et al.* published a groundbreaking study titled “Intestinal CXCR6+ILC3s Migrate to the Kidney and Promote Renal Fibrosis *via* PD-1-mediated Activation” in *Immunity*, providing novel insights into the gut-kidney migration pathway and pathogenic mechanisms of ILC3s in renal fibrosis [4]. This study holds significant implications for future research on the gut-kidney axis and the development of targeted therapies.

### 3.5 Hotspot Keyword Analysis

Keywords are a concise representation of the core content of articles (Table **5**), and their frequency and co-occurrence density can reflect research hotspots and trends in a field. A total of 20,847 keywords were analyzed, indicating the breadth and diversity of research topics in this field. Through the analysis of keyword timeline diagram (Fig. **6A**) and keyword co-occurrence density diagram (Fig. **6D**), after removing synonyms, the most frequently occurring keywords were expression (1,266 times), natural-killer-cells (928 times), innate lymphoid-cells (884 times), T-cells (830 times), dendritic cells (744 times), inflammation (739 times), activation (612 times), regulatory t-cells (546 times), and immunotherapy (534 times]. Cluster analysis (Fig. **6C**) identified four main clusters: “#0 immunotherapy”, “#1 inflammatory bowel disease”, “#2 differentiation”, and “#3 expression”. The modularity Q value was 0.3663, and the mean silhouette value was 0.679, indicating robust and reliable clustering [20]. Combined with high-frequency keyword analysis, inflammatory pathological mechanisms and immunotherapy remain key themes in ILC research in the gut and kidney.

Burst analysis revealed the most prominent research methods and topics in the field (Fig. **6B**). The keywords monoclonal antibody and carcinoma had the longest burst durations, while *in vivo* exhibited the highest burst strength. The research focus has shifted from early topics such as renal cell carcinoma, monoclonal antibody, and intraepithelial lymphocytes to more recent topics like tumor microenvironment and commensal bacteria. This suggests that future research may further explore the interactions between the microbiome and the host immune system in the tumor microenvironment, as well as the development of targeted therapeutic strategies.

### 3.6 Co-Cited Reference Analysis

The included articles cited a total of 275,297 references. Table **6** and Fig. (**7A**) summarize the top 10 most cited references, which include 3 review articles and 7 original research studies. The most cited article was the 2018 review “Innate Lymphoid Cells: 10 Years On” by Eric Vivier, published in *Cell* [21]. This article systematically summarizes the biological functions of ILCs, revealing their roles beyond traditional immunity (*e.g.*, metabolic regulation, tissue repair, and neural interactions). It propelled ILC research from immune defense to multi-system integration (*e.g.*, the metabolic-immune-neural axis), providing a theoretical framework for ILC-targeted disease therapies and profoundly influencing the field. The second most cited article, by Hergen Spits, proposed a unified nomenclature for ILCs (classified into three groups based on cytokines and transcription factors) [3]. This standardized classification accelerated ILC research and laid the foundation for subsequent functional studies and clinical translation. The third most cited article, by Sung *et al.*, reported on the global burden of cancer [22], providing epidemiological support for ILC research in tumor microenvironments (*e.g.*, intestinal cancer). These highly cited articles primarily focus on the foundational theories of ILCs, the gut-microbiota-immune axis, and inflammation and disease mechanisms. Their publication timeline reflects the evolution of ILC research from basic classification to multi-dimensional exploration of mechanisms, diseases, and therapies.

Cluster analysis of co-cited references (Fig. **7B**) identified eight clusters with a modularity Q value of 0.5811 and a mean silhouette value of 0.8966. The clusters were: “#0 tissue repair”, “#1 tumor necrosis factor”, “#2 colorectal cancer progression”, “#3 innate lymphoid cell”, “#4 treatment-induced cancer rejection”, “#5 host-microbiota interaction,” “#6 colon adenocarcinoma”, and “#7 single-cell transcriptome analysis”. These clusters indicate a broad scope of research, ranging from basic immune mechanisms (*e.g.*, characteristics of innate lymphoid cells) to clinical applications (*e.g.*, treatment-induced cancer rejection), highlighting the importance of interdisciplinary collaboration in microbiology, immunology, and oncology. Burst analysis of citations (Fig. **7C**) revealed that Eric Vivier’s article had the highest burst strength [21], while articles by Hergen Spits and Stephanie L [3, 23]. Sanos had the longest burst durations, underscoring their significant influence on the field. Stephanie L. Sanos’ study discovered that commensal microbiota regulate the differentiation of RORγt^+^IL-22^+^ cells to maintain gut homeostasis, providing a reliable basis for targeting the IL-22 pathway in treating mucosal inflammation or infectious diseases.

## 4 DISCUSSION

This study, through a bibliometric analysis of research on innate lymphoid cells (ILCs) in the gut and kidney from 2004 to 2024, reveals global research trends in this field. Over the past two decades, ILC-related research has shown significant growth, peaking in 2024 with 622 publications, indicating sustained high academic interest. Geographically, the United States (2,145 publications), China (1,539 publications), and Germany (679 publications) ranked as the top three contributors in terms of publication volume. However, the research influence varied significantly. The United States stood out with the highest citation count (159,978 citations) and a robust institutional collaboration network (*e.g.*, close collaboration between Harvard Medical School and the National Cancer Institute), reflecting its long-standing foundation in basic research and interdisciplinary collaboration. Although France did not rank among the top three in publication volume, it achieved the highest average citation frequency (85.06 citations per article), indicating superior research quality. Despite China’s high publication output, its average citation frequency remained relatively low (26.2 citations per article). Optimizing research resource allocation and strengthening international collaborations may be key strategies for enhancing China’s research influence in the future.

In terms of institutional and author contributions, Harvard Medical School and the French National Institute of Health and Medical Research (Inserm) emerged as the most influential institutions, publishing 101 and 96 articles, respectively, with average citation counts exceeding 100. Their research has laid a critical foundation for understanding the functions of ILCs in the gut and kidney. Additionally, Gregory F. Sonnenberg and David Artis were identified as the most influential scholars in the field. Their work focuses on the relationships between ILCs and gut microbiota, inflammatory bowel disease (IBD), and renal fibrosis, providing pivotal insights into the pathological mechanisms and therapeutic strategies involving ILCs.

Keyword and co-cited journal analyses highlighted research hotspots and trends in the field of ILCs in the gut and kidney. Keyword clustering identified “inflammatory bowel disease” and “immunotherapy” as top priorities, underscoring the growing interest in the application of ILCs in inflammatory diseases and immunotherapies. Co-cited journal analysis further supported this trend, with *Immunity* and *Nature* being the most frequently cited journals, reflecting their significant influence in ILC research. Notably, *Immunity*, with its high citation frequency and impact factor (IF=25.5), holds a prestigious position in immunology. Recent advances have emphasized the functional heterogeneity and regulatory networks of ILCs [24], offering new perspectives on the pathological mechanisms and intervention strategies for IBD. For instance, ILC3s maintain epithelial barrier integrity in the gut through RORγt-dependent IL-22 secretion, which promotes antimicrobial peptide production and mucosal repair [25]. However, in IBD, dysbiosis and chronic inflammation drive ILC3s toward a pro-inflammatory phenotype characterized by excessive secretion of IL-17A and granulocyte-macrophage colony-stimulating factor (GM-CSF), disrupting immune tolerance and exacerbating tissue damage [25, 26]. Similarly, ILC2s contribute to IBD through cytokine stimulation and their ability to maintain the intestinal barrier [25]. ILC2-derived IL-13 triggers the differentiation of intestinal stem cells into Tuft cells and goblet cells, facilitating tissue repair [27]. However, Camelo *et al.* found that IL-13+ILC2s were increased in a mouse model of oxazolone-induced colitis, suggesting a potential pro-inflammatory role for ILC2s in ulcerative colitis (UC) [28].

Numerous studies have also demonstrated the role of ILCs in the pathophysiology of kidney diseases, particularly ILC3s. Renal fibrosis, a common pathological process in chronic kidney disease (CKD) progressing to end-stage kidney disease (ESKD) [29], is characterized by excessive deposition of extracellular matrix (ECM) in renal tissues. Recent studies have shown that CXCR6+ ILC3s in the gut can migrate to the kidney *via* the CXCL16/CXCR6 chemotactic axis [4]. Through PD-1 on ILC3s, which inhibits IL-23R endocytosis, the JAK2/STAT3 pathway is continuously activated, promoting IL-17A secretion and driving renal fibrosis. This discovery provides new insights into preventing renal fibrosis by targeting the gut-kidney axis. Conversely, ILC2s exhibit protective effects. Nagashima *et al.* reported that IL-33-stimulated renal ILC2s inhibit myofibroblast transdifferentiation through IL-13, attenuating adenine-induced fibrosis [30]. Similarly, IL-33 pretreatment enriched renal ILC2s and regulatory T cells (Tregs), reducing fibrosis in a unilateral ureteral obstruction (UUO) model [31]. These findings highlight the duality of ILC subsets: while ILC3s exacerbate fibrosis, ILC2s may offer reparative functions. In lupus nephritis (LN), dysregulated ILC activity contributes to systemic autoimmunity. Wang *et al.* observed elevated circulating ILC1s and reduced ILC2s in LN patients, correlating with leukocytosis and hypocomplementemia. IFN-γ-driven ILC1 polarization may exacerbate renal inflammation, while ILC2 deficiency impairs tissue repair [32].

In the field of immunotherapy, targeting the regulatory networks of ILCs has emerged as a promising direction. In diabetic nephropathy (DN), mesenchymal stromal cells (ORBCEL-M) modulate NK T cells and inflammatory monocytes, stabilizing eGFR decline [33]. Similarly, rosmarinic acid slows DN progression by inhibiting oxidative stress in ILCs and macrophages [34]. The PD-1/IL-23R signaling axis in ILC3s has been identified as a critical node in fibrosis progression. Zhou *et al.* demonstrated that the CXCL16/CXCR6 chemotactic axis and PD-1 blockade could reverse the process by which gut-derived ILC3s promote IL-17A secretion and exacerbate fibrosis [4]. Similar mechanisms are evident in intestinal inflammation: Song *et al.* successfully alleviated DSS-induced colitis in mice using a PD-L1-Fc fusion protein, confirming the central role of the PD-1 pathway in suppressing excessive immune responses in the gut [35]. Furthermore, microbial metabolites regulating ILCs offer new avenues for precision therapy. Su *et al.* revealed that gut microbiota-derived tryptophan metabolites (*e.g.*, indolepropionic acid) activate ILC3s through the aryl hydrocarbon receptor (AHR), promoting IL-22 secretion to maintain gut homeostasis [36].

Burst analysis indicates that the research focus has gradually shifted from early topics such as “renal cell carcinoma” and “monoclonal antibody” to “tumor microenvironment” and “commensal bacteria”, suggesting that future studies may further explore the interactions between ILCs and the microbiome in the tumor microenvironment and their potential applications in disease treatment.

Recent studies have shown that ILCs are emerging as key regulators of tumor immunity. In renal cell carcinoma (RCC), different ILC subsets exhibit context-dependent roles. While ILC1s and natural killer cells are associated with anti-tumor responses, dysregulated ILC3 activity may promote immunosuppression through IL-15 signaling [37, 38]. A recent study by Amisaki *et al.* revealed that IL-33-activated ILC2s can induce the formation of tertiary lymphoid structures (TLS) in pancreatic cancer *via* lymphotoxin (LT) signaling, enhancing anti-tumor immune responses [39]. This “lymphoid neogenesis” capability is linked to the gut origin of ILC2s and their microbiome-dependent migration, providing new targets for immunotherapy in IBD-associated tumors. However, Jou *et al.* found that IL-25-secreting intestinal ILC2s can promote a pro-tumor niche by recruiting myeloid-derived suppressor cells (MDSCs), potentially contributing to colorectal cancer (CRC) pathogenesis, highlighting the plasticity of these cells [40].

The influence of gut commensal bacteria on ILCs is another area of growing interest. As mentioned earlier, Amisaki *et al.* proposed that the gut microbiota-dependent migration of ILC2s in pancreatic cancer enhances anti-tumor immune responses [39]. Additionally, Fusobacterium nucleatum and Helicobacter hepaticus modulate ILC3 activity, with the latter inducing TLS formation through microbiota-specific T follicular helper (Tfh) cells, enhancing CD8+ T and NK cell infiltration [41, 42]. These findings underscore the impact of the microbiome on ILC-mediated immunity, suggesting that prebiotic or probiotic strategies could modulate anti-tumor ILC activity.

Innovative therapies are leveraging the plasticity of ILCs. In CRC, IL-15-enhanced chimeric antigen receptor (CAR)-NK cells targeting CD70 have been shown to eliminate tumor cells and cancer-associated fibroblasts (CAFs) [43]. For RCC, Zhai *et al.* found that high expression of ALKBH5 is associated with poor prognosis in CRC [44], correlating with the accumulation of MDSCs and a reduction in NK cells and cytotoxic CD8+ T cells. Encapsulating ALKBH5 small interfering RNA (siRNA) in vesicular nanoparticles enhances the efficacy of anti-PD-1 therapy, making ALKBH5 targeting a potential strategy to sensitize CRC to immunotherapy.

Despite significant progress, the clinical translation of ILCs faces challenges, including off-target effects on adaptive immunity [45], variable patient responses due to ILC heterogeneity, and the predominance of preclinical studies on microbiome-ILC interactions. Species differences in animal models further limit the clinical applicability of these findings. Future research should integrate artificial intelligence predictive models and single-cell multi-omics to dissect the dynamic networks of ILCs, while exploring combined strategies such as ILC adoptive transfer and gene editing to bridge the gap from mechanisms to clinical applications.

This study’s strength lies in its systematic use of bibliometric and visual analysis methods to summarize the current research landscape of ILCs in the gut and kidney, revealing research hotspots and developmental trends. The data were sourced from the Web of Science Core Collection (WOSCC), ensuring the authority and comprehensiveness of the literature. Additionally, the multi-dimensional analysis using VOSviewer and CiteSpace provided not only macro-level data on publication volume, international collaborations, and institutional contributions but also micro-level insights into keywords and co-cited journals, offering valuable references for future research.

However, this study has several limitations. First, the literature search was confined to the WOSCC database, potentially omitting significant studies published in non-WOSCC-indexed journals. Second, bibliometric analysis primarily relies on quantitative data, which may not fully reflect the quality and innovation of the research. Furthermore, this study was limited to English publications and did not account for variations in publication quality, which may affect the comprehensiveness of the analysis. Given the rapid evolution of the field, this study may not fully capture the latest developments and breakthroughs. Future research should consider integrating data from additional sources and incorporating the most recent findings to enhance the accuracy of conclusions.

## CONCLUSION

In summary, this study systematically summarizes the current research status and developmental trends of ILCs in the gut and kidney through bibliometric and visual analysis. Research hotspots focus on inflammatory mechanisms, immunotherapy, and the interactions between ILCs and the microbiome, while future studies may further explore the functions and regulatory mechanisms of ILCs in the tumor microenvironment. These findings provide theoretical support for the application of ILCs in disease treatment and highlight the importance of interdisciplinary collaboration (*e.g.*, immunology, microbiology, and oncology) in advancing the field. However, balancing the pro-repair and pro-fibrotic functions of ILC3s remains a core challenge for future research. Through technological innovation and interdisciplinary approaches, the transition from “mechanistic mapping” to “clinical translation” can be achieved, ultimately driving breakthrough applications of ILC-targeted therapies in gut and kidney diseases. Future research should strengthen international collaborations to produce high-quality studies and further explore the specific mechanisms and clinical potential of ILCs in the gut-kidney axis.

## Figures and Tables

**Fig. (1):**
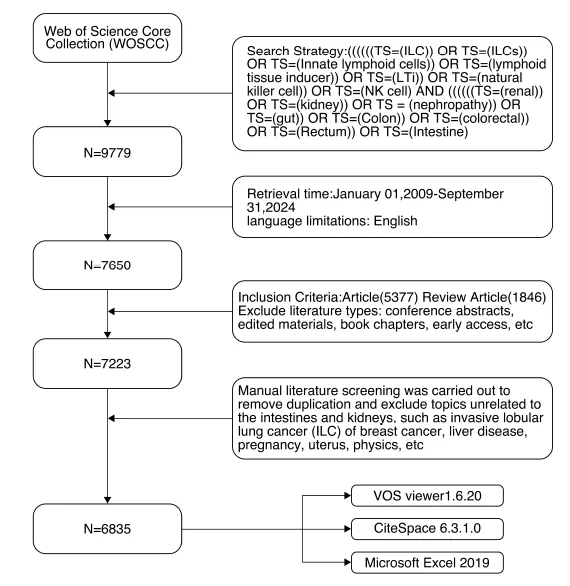
Flowchart of data extraction.

**Fig. (2):**
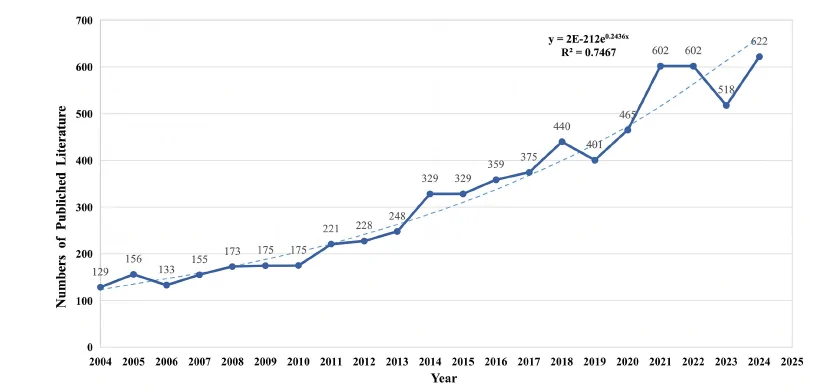
Annual distribution of publications in the field.

**Fig. (3):**
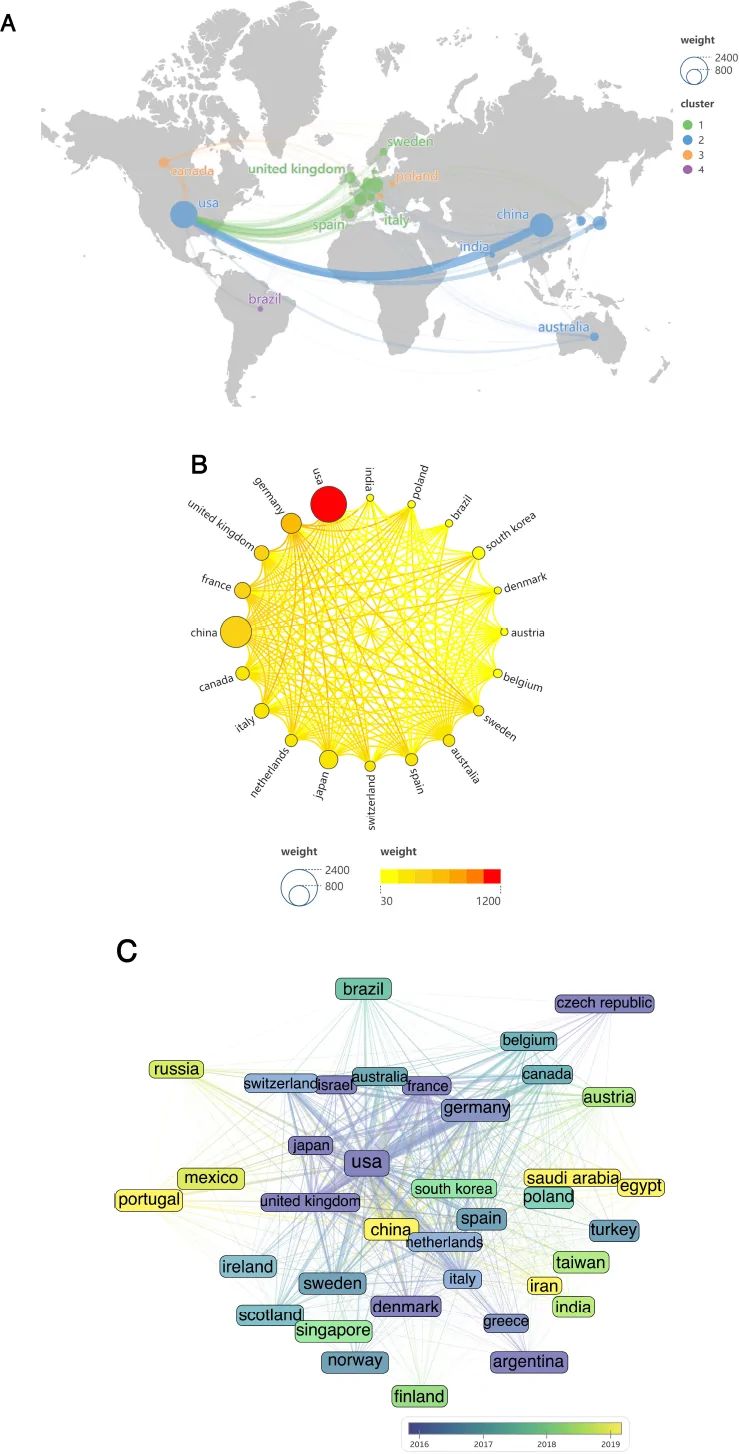
(**A**) Global distribution of publications by country; (**B**) Collaboration network among countries/regions; (**C**) Temporal visualization of research contributions by country/region.

**Fig. (4):**
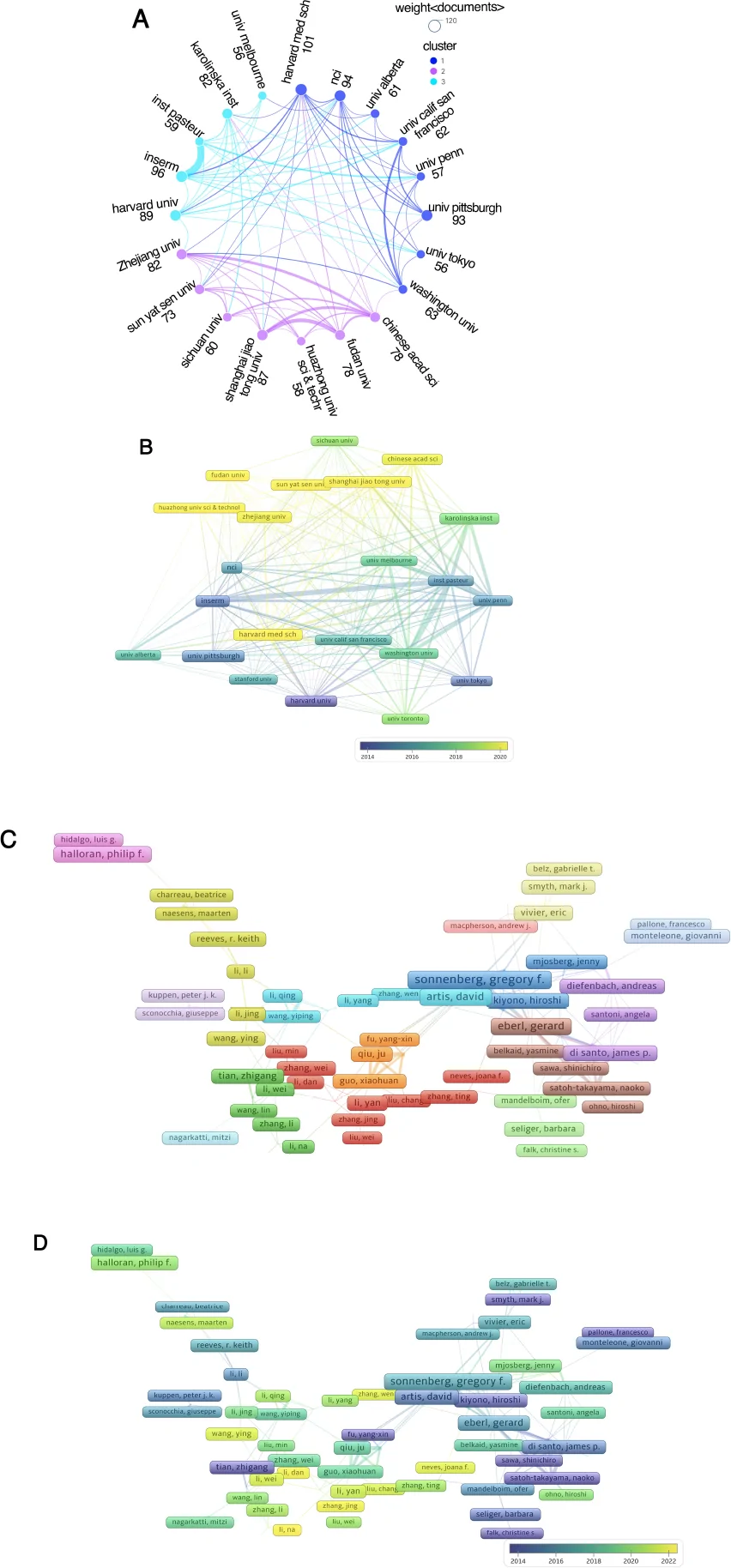
Visualization of research institutions and contributing authors in the field. (**A**) Collaboration network of research institutions (different colors represent clusters). (**B**) Temporal visualization of institutional collaborations. (**C**) Collaboration network of contributing authors (different colors represent clusters). (**D**) Temporal visualization of author collaborations.

**Fig. (5):**
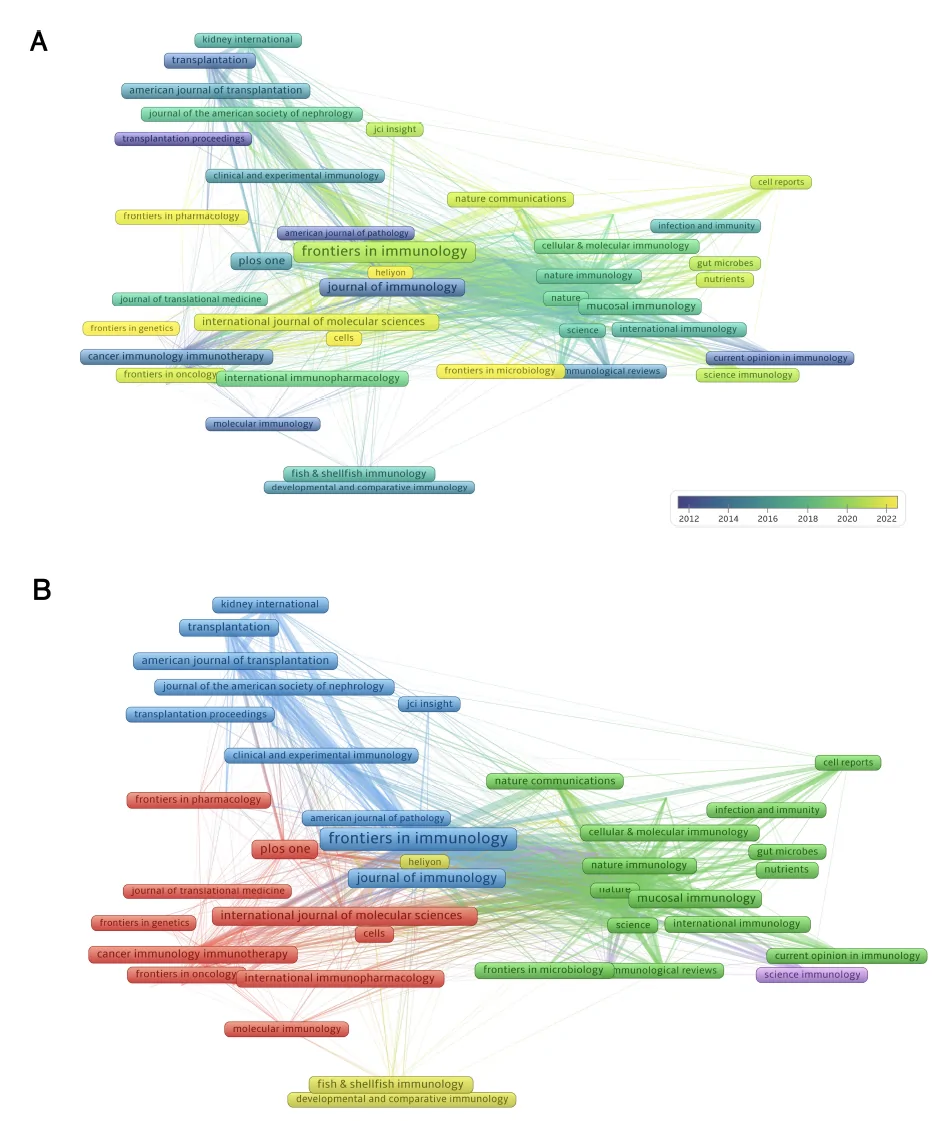
Analysis of journal publications in the field. (**A**) Cluster visualization of major journals. (**B**) Temporal visualization of journal publications.

**Fig. (6):**
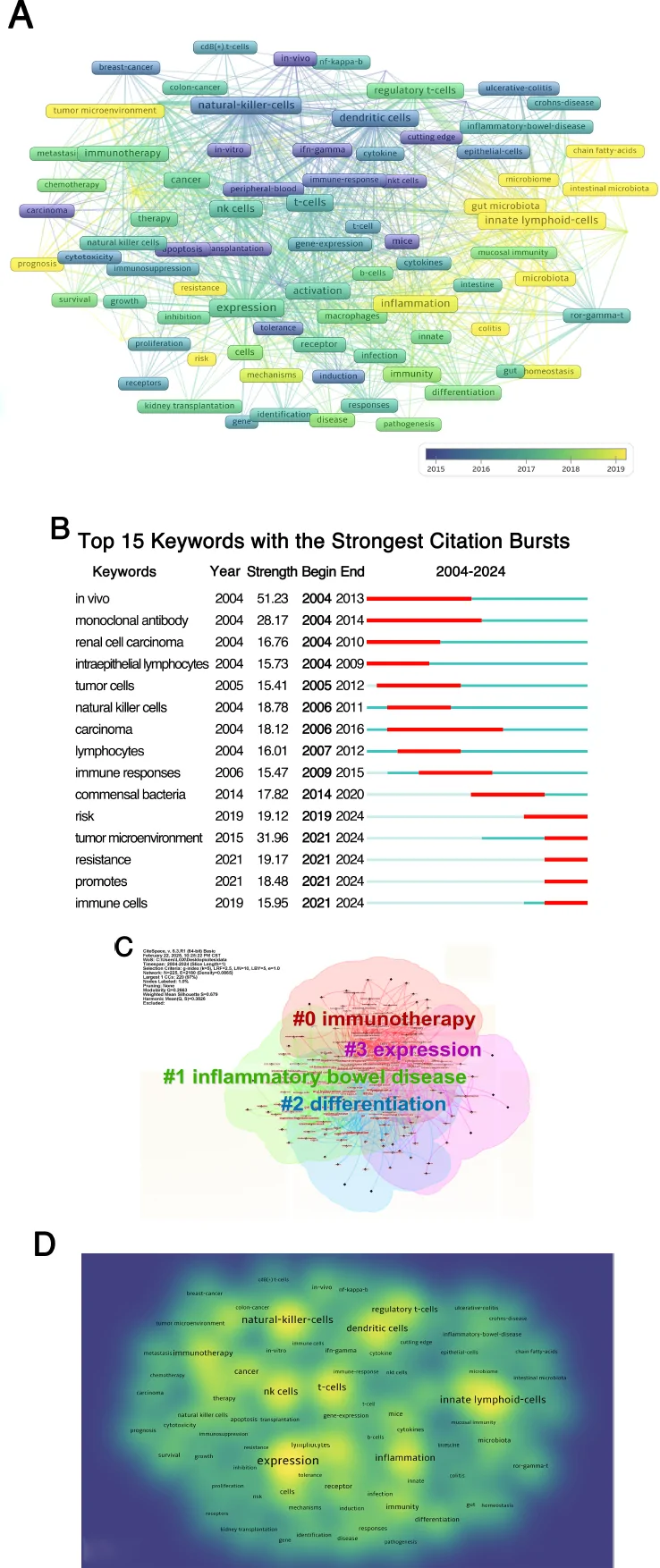
Keyword clustering and burst analysis. (**A**) Timeline visualization of keywords. (**B**) Top 15 burst keywords. (**C**) Cluster analysis of keywords. (**D**) Keyword density map (brightness proportional to keyword frequency).

**Fig. (7):**
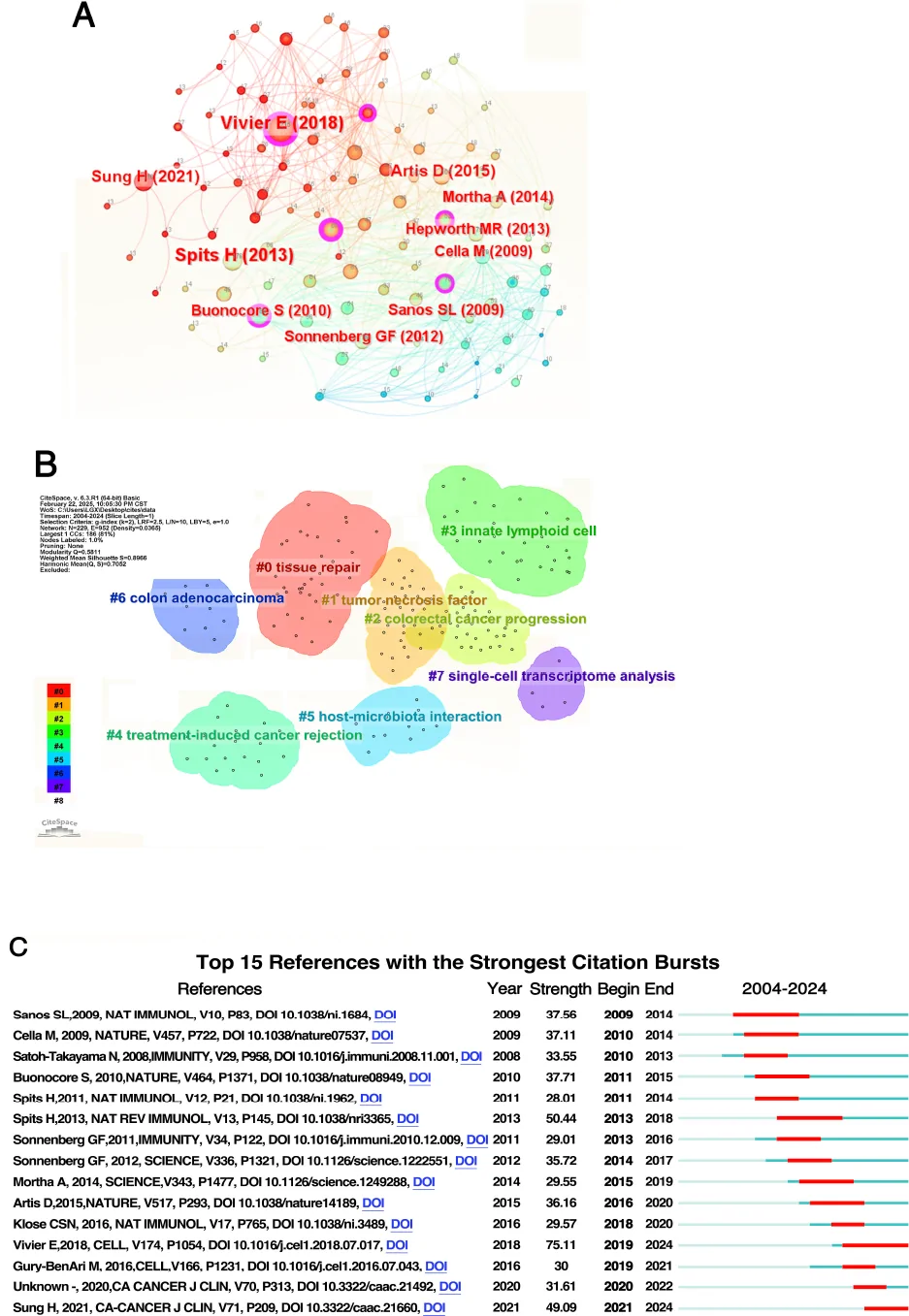
Co-cited journal visualization analysis. (**A**) Network visualization of co-cited journals (highlighting top 10 cited journals). (**B**) Cluster analysis of co-cited journals. (**C**) Top 15 burst journals.

**Table 1:** Top 5 countries/regions in terms of publication volume and citation frequency.

Rank	Country	Publications	Citations	Average Citation/Publication
1	USA	2145	159978	74.58
2	China	1539	40323	26.20
3	Germany	679	41773	61.52
4	Japan	572	22467	39.28
5	France	432	36745	85.06

**Table 2:** Top 10 institutions by publication volume.

Rank	Organizations	Publications	Citations	Average Citation/Publication
1	Harvard Medical School	101	5688	56.32
2	Inserm	96	12105	126.09
3	NCI	94	9154	97.38
4	University of Pittsburgh	93	6519	70.10
5	Harvard University	89	8963	100.71
6	Shanghai Jiao tong University	87	3174	36.48
7	Zhejiang University	82	2419	29.50
8	Karolinska Institute	82	4816	58.73
9	Chinese Academy of Sciences	78	3876	49.69
10	Fudan University	78	1454	18.64

**Table 3:** Top 10 authors by publication volume.

Rank	Authors	Documents	Citations	Average Citation/Publication
1	Gregory F Sonnenberg	30	5471	182.37
2	Artis, David	25	8201	328.04
3	Eberl, Gerard	25	4402	176.08
4	Halloran, Philip F	21	1243	59.19
5	Colonna, Marco	19	2881	151.63
6	Di Santo, James P	19	2552	134.32
7	Kiyono, Hiroshi	19	1759	92.58
8	Hepworth, Matthew R	17	2256	132.71
9	Qiu, Ju	17	1783	104.88
10	Zhou, Liang	17	1914	112.59

**Table 4:** Top 10 source journals by publication volume.

Rank	Source	Publications	Citations	Average Citation/Publication	IF (2023)
1	Frontiers in Immunology	460	17612	38.29	5.7
2	Journal of Immunology	174	10218	58.72	3.6
3	Plos one	142	4712	33.18	2.9
4	International Journal of Molecular Sciences	113	3220	28.50	4.9
5	Mucosal Immunology	80	3978	49.73	8.1
6	Scientific Reports	74	1449	19.58	3.7
7	Fish & Shellfish Immunology	69	2145	31.09	4.1
8	Immunity	67	14250	212.69	25.5
9	Transplantation	67	1879	28.04	5.5
10	Cancer Immunology Immunotherapy	66	1942	29.42	4.5

**Table 5:** Top 10 high frequency cited journals.

Rank	Source	Citations	IF (2023)
1	Immunity	15148	25.5
2	Journal of Immunology	21656	3.6
3	Nature	14693	50.5
4	JOURNAL of Experimental Medicine	12608	12.8
5	Nature Reviews Immunology	10624	27.8
6	SCIENCE	10837	44.8
7	Proceedings of the National Academy of Sciences of the United States of America	10621	9.4
8	Cell	8273	45.6
9	Frontiers in Immunology	8131	5.7
10	Blood	8138	21.1

**Table 6:** Top 10 cited references.

Rank	Title	Type	Citations	Journal	IF (2023)	Corresponding Author	Affiliation	Year
1	Innate lymphoid cells: 10 Years On	Review	215	Cell	45.5	Eric Vivier	Aix Marseille University	2018
2	Innate lymphoid cells-a proposal for uniform nomenclature	Review	178	Nature Reviews Immunology	67.7	Hergen Spits	University of Amsterdam	2013
3	Global Cancer Statistics 2020: GLOBOCAN Estimates of Incidence and Mortality Worldwide for 36 Cancers in 185 Countries	Article	116	Ca-a Cancer Journal For Clinicians	503.1	Hyuna Sung	American Cancer Society	2021
4	The biology of innate lymphoid cells	Review	105	Nature	50.5	David Artis	Cornell University	2015
5	Innate lymphoid cells promote anatomical containment of lymphoid-resident commensal bacteria	Article	101	Science	44.7	Gregory F Sonnenberg	University of Pennsylvania	2012
6	Innate lymphoid cells drive interleukin-23-dependent innate intestinal pathology	Article	89	Nature	50.5	Sofia Buonocore	University of Oxford	2010
7	Innate lymphoid cells regulate CD4+ T-cell responses to intestinal commensal bacteria	Article	88	Nature	50.5	Matthew R Hepworth	University of Pennsylvania	2013
8	Microbiota-dependent crosstalk between macrophages and ILC3 promotes intestinal homeostasis	Article	86	Science	44.7	Arthur Mortha	Mount Sinai Hospital	2014
9	A human natural killer cell subset provides an innate source of IL-22 for mucosal immunity	Article	85	Nature	50.5	Marina Cella	Washington University	2009
10	RORgammat and commensal microflora are required for the differentiation of mucosal interleukin 22-producing NKp46+ cells	Article	82	Nature Immunology	27.7	Stephanie L Sanos	University of Freiburg	2009

## LIST OF ABBREVIATIONS

AHR: Aryl Hydrocarbon Receptor
CAFs: Cancer-associated Fibroblasts
CAR: Chimeric Antigen Receptor
CKD: Chronic Kidney Disease
CRC: Colorectal Cancer
CTLA4: Cytotoxic T-lymphocyte-associated Protein 4
DN: Diabetic Nephropathy
ECM: Extracellular Matrix
ESKD: End-Stage Kidney Disease
GM-CSF: Granulocyte-Macrophage Colony-Stimulating Factor
IBD: Inflammatory Bowel Disease
IELs: Intraepithelial Lymphocytes
IF: Impact Factor
IFN-γ: Interferon-Gamma
IL-23: Interleukin-23
ILC: Innate Lymphoid Cell
Inserm: French National Institute of Health and Medical Research
JAK2: Janus Kinase 2
LLR: Log-Likelihood Ratio
LN: Lupus Nephritis
LT: Lymphotoxin
MDSCs: Myeloid-Derived Suppressor Cells
MNPs: Mononuclear Phagocytes
NCI: National Cancer Institute
NK: Natural Killer Cell
ORBCEL-M: Mesenchymal Stromal Cell Product
PD-1: Programmed Cell Death Protein 1
RCC: Renal Cell Carcinoma
SCI-EXPANDED: Science Citation Index Expanded
SSCI: Social Sciences Citation Index
STAT3: Signal Transducer and Activator of Transcription 3
TFH: T Follicular Helper Cell
TLS: Tertiary Lymphoid Structures
TREGS: Regulatory T Cells
UUO: Unilateral Ureteral Obstruction
WOSCC: Web of Science Core Collection
